# Supplementary material for: Coupled feedback loops maintain synaptic long-term potentiation: A computational model of PKMzeta synthesis and AMPA receptor trafficking
Source: PLoS Comput Biol. 2018 May 29;14(5):e1006147. doi: 10.1371/journal.pcbi.1006147 (PMC5993340; doi:10.1371/journal.pcbi.1006147)
Supplement: S1 Text — (DOCX) [file pcbi.1006147.s001.docx]

# S1 Text. Estimating the number of PKMζ molecules in a spine head

Zeta-inhibitory peptide (ZIP) disrupts the ability of PKMζ to potentiate synaptic transmission when applied extracellularly in concentrations around 1 μM [1]. According to Serrano et al. [2], 5 μM completely blocks potentiation and 1 μM reduces the increase in AMPAR response by 50%.

Assuming that ZIP molecules enter neurons by diffusion only (i.e. they are not actively transported into cells), 1 μM would be an upper limit on the intracellular ZIP concentration.

1 μM = 10^-6^ * 6.022 * 10^23^ molecules/liter = 10^-6^ * 6.022 * 10^23^ * 10^3^ ≈ 6 *10^20^ molecules/m^3^.

The volume of a dendritic spine head is between 0.01 and 0.1 μm^3^ [3,4]. If we conservatively use the upper limit of this range, 10^-1^ μm^3^ = 10^-19^ m^3^, then the maximum number of ZIP molecules that would be present in a spine head due to a 1 μM bath concentration would be ≈ 6 *10^20^ * 10^-19^ = 60.

Because ZIP’s inhibitory action results from ZIP molecules binding to PKMζ molecules in a one-to-one ratio, we may assume that for ZIP to significantly disrupt PKMζ activity, the number of ZIP molecules must be at least of the same order of magnitude as the number of PKMζ molecules.

Consequently, the number of PKMζ molecules at a potentiated synapse may be estimated to be fewer than about one hundred.

# References

1. Yao Y, Shao C, Jothianandan D, Tcherepanov A, Shouval H, Sacktor TC. Matching biochemical and functional efficacies confirm ZIP as a potent competitive inhibitor of PKMζ in neurons. Neuropharmacology. 2013;64: 37–44. doi:10.1016/j.neuropharm.2012.07.018

2. Serrano P, Yao YD, Sacktor TC. Persistent phosphorylation by protein kinase M zeta maintains late-phase long-term potentiation. J Neurosci. 2005;25: 1979–1984. doi:10.1523/JNEUROSCI.5132-04.2005

3. Bartol TM, Bromer C, Kinney JP, Chirillo MA, Bourne JN, Harris KM, et al. Hippocampal spine head sizes are highly precise. bioRxiv. 2015; 016329. doi:10.1101/016329

4. Kasthuri N, Hayworth KJ, Berger DR, Schalek RL, Conchello JA, Knowles-Barley S, et al. Saturated reconstruction of a volume of neocortex. Cell. 2015;162: 648–661. doi:10.1016/j.cell.2015.06.054
